# Subsurface temperature estimates from a Regional Ocean Modelling System (ROMS) reanalysis provide accurate coral heat stress indices across the Main Hawaiian Islands

**DOI:** 10.1038/s41598-024-56865-x

**Published:** 2024-03-19

**Authors:** Jessica N. Perelman, Kisei R. Tanaka, Joy N. Smith, Hannah C. Barkley, Brian S. Powell

**Affiliations:** 1 Cooperative Institute for Marine and Atmospheric Research, University of Hawaii, 1000 Pope Road, Honolulu, HI 96822 USA; 2 Pacific Islands Fisheries Science Center, National Marine Fisheries Service, 1845 Wasp Boulevard, Honolulu, HI 96818 USA; 3 Department of Oceanography, University of Hawaiʻi at Mānoa, Honolulu, HI 96822 USA

**Keywords:** Coral reef, Main Hawaiian Islands, Regional Ocean Modelling System (ROMS), Skill assessment, Bleaching, Ecology, Ocean sciences, Marine biology, Physical oceanography

## Abstract

As ocean temperatures continue to rise, coral bleaching events around the globe are becoming stronger and more frequent. High-resolution temperature data is therefore critical for monitoring reef conditions to identify indicators of heat stress. Satellite and in situ measurements have historically been relied upon to study the thermal tolerances of coral reefs, but these data are quite limited in their spatial and temporal coverage. Ocean circulation models could provide an alternative or complement to these limited data, but a thorough evaluation against in situ measurements has yet to be conducted in any Pacific Islands region. Here we compared subsurface temperature measurements around the nearshore Main Hawaiian Islands (MHI) from 2010 to 2017 with temperature predictions from an operational Regional Ocean Modeling System (ROMS) to evaluate the potential utility of this model as a tool for coral reef management. We found that overall, the ROMS reanalysis presents accurate subsurface temperature predictions across the nearshore MHI region and captures a significant amount of observed temperature variability. The model recreates several temperature metrics used to identify coral heat stress, including predicting the 2014 and 2015 bleaching events around Hawaiʻi during the summer and fall months of those years. The MHI ROMS simulation proves to be a useful tool for coral reef management in the absence of, or to supplement, subsurface and satellite measurements across Hawaiʻi and likely for other Pacific Island regions.

## Introduction

The thermal environment around Hawaiʻi plays a critical role in coral reef ecosystems as elevated seawater temperatures are a primary driver of coral stressors such as bleaching^1^. This phenomenon leaves corals vulnerable to disease and mortality, and mass bleaching events around the globe are increasing in frequency due to warming ocean temperatures^2,3^. Thus, understanding the temperature variability experienced by corals at depth is important for understanding and predicting their responses to changing ocean conditions. Historically, scientists and resource managers have relied on in situ temperature measurements and satellite-derived sea surface temperatures (SST) to study the thermal tolerances of coral reefs around Hawaiʻi^4,5^. However, the spatiotemporal resolution of these measurements are quite limited; satellite data measures only a thin layer (“skin”) of surface waters and is limited to nightly temperatures, and in situ measurements are limited by the locations, depths, and times that they record data.

Ocean circulation models could provide an alternative or complementary approach to track surface and subsurface temperatures across nearshore habitats at much higher spatial and temporal resolutions. One such model is the Regional Ocean Modelling System (ROMS^6^), a free-surface, terrain-following data assimilative ocean circulation model. ROMS is widely used by the scientific community to investigate both marine and freshwater circulation patterns ^7–9^ and is supported by global contributors. For the Main Hawaiian Islands (MHI) region, ROMS has been implemented and maintained operationally by the Pacific Islands Ocean Observing System (PacIOOS) and provides temperature, salinity, and currents in four dimensions. The boundary conditions for ROMS are taken from the Hybrid Coordinate Ocean Model (HYCOM) at 1/12 degree and are specified daily. Every day, the model uses four-dimensional variational data assimilation (4D-Var) to incorporate the previous 3 days of available observations and produces a 7 day forecast^10^. A local, hi-resolution, Weather Regional Forecast (WRF) provides the atmospheric forcing at approximately 6 km resolution, but the data assimilation alters the initial conditions, lateral forcing, and surface forcing in the model. A 10-year reanalysis of the MHI using ROMS was produced for the MHI by assimilating observations from surface measurements (satellite and high frequency radar) and depth profiles (Argo, Seagliders, and shipboard CTD^11^. We refer to these reanalysis data as MHIA. In order to understand how well the MHIA reflects nearshore subsurface temperatures, a thorough evaluation against in situ measurements must be conducted across the MHI region.

Here we present a comprehensive skill assessment of MHIA subsurface temperatures for the MHI region to evaluate the reliability and biases of this reanalysis and its potential use as a tool for coral reef management. For comparison, we used in situ temperature data collected by subsurface temperature recorders (STR) that monitor thermal conditions at nearshore reef locations around the MHI. While ROMS has been previously assessed in other regions and ecosystems^12^, this is the first comprehensive evaluation of MHIA temperature data in the Pacific Islands region. We aim to assess the feasibility of using MHIA as a tool for nearshore subsurface temperature monitoring for coral reef management. Importantly, we evaluate how accurately MHIA estimates temperature variability across several metrics used to identify heat stress in corals, including extreme heat or cold events. Given that this ocean model is intended to reflect offshore circulation patterns at a broader scale than point-source measurements, we expect to see significant bias in the modeled temperatures with respect to observations. This study provides evidence-based guidelines that can be used to promote the utility of numerical ocean circulation models for enhanced reef studies and management actions.

## Methods

### In situ subsurface temperature data collection

**Figure 1:**
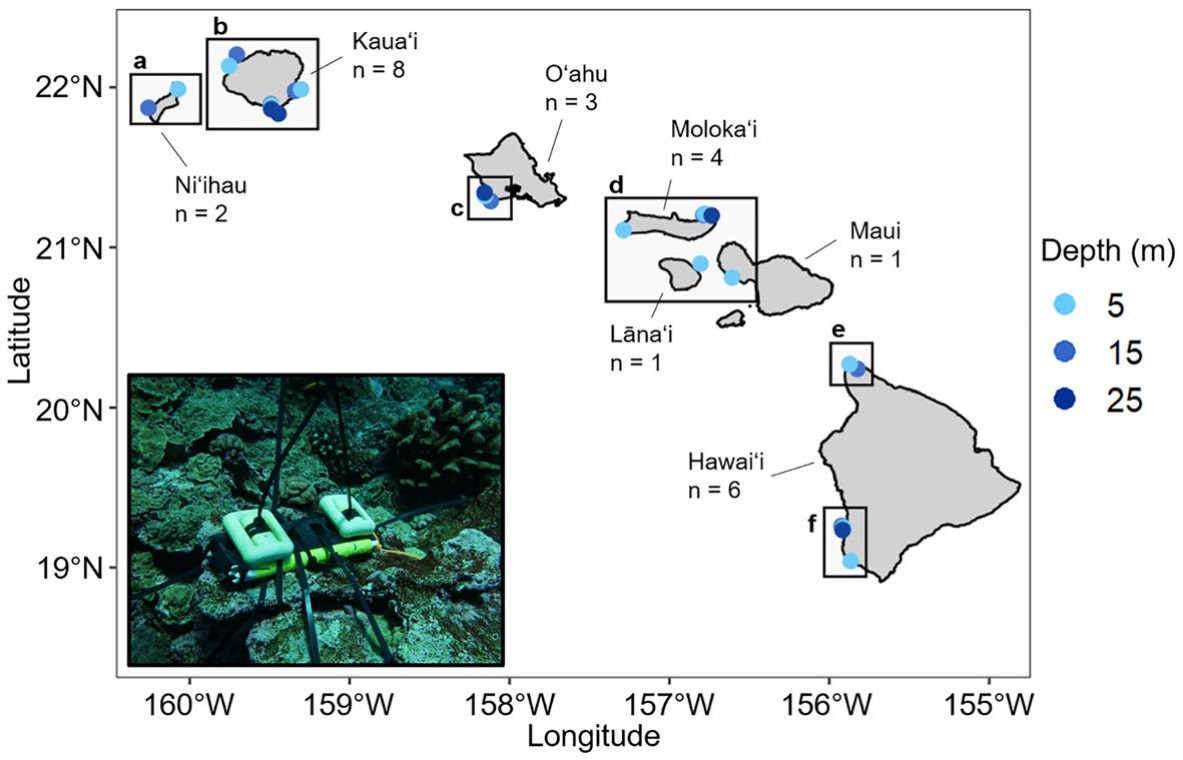
Map of STR locations around the Main Hawaiian Islands (MHI) with depth ranges and number of sites (n) indicated for each island. Letters correspond to panels in Fig. 3. Inset: photo of an STR deployed on reef (credit: Joy Smith). MHIA temperature data extraction and pairing.

Subsurface temperature recorders (STR; Sea-Bird SBE56 temperature sensors; typical stability 0.0002 °C month^−1^ or 0.002 °C year^−1^; initial accuracy ± 0.002 °C) were deployed at 25 locations across the MHI from Oct 2010 to May 2017 to monitor the thermal environment around coral reefs (Fig. 1; Supplementary Table S1). STRs initially deployed in 2010 were recovered every 3 years and newly serviced STRs with fresh batteries were re-deployed in the same exact location to obtain a continuous time series. Unfortunately, battery failure for several instruments during the 2013 deployment led to gaps in the time series data. The STRs were deployed and recovered as part of NOAA’s National Coral Reef Monitoring Program (NCRMP) at fixed-sites at depths approximately 5 m, 15 m, and 25 m. This in situ data covers roughly the upper third of coral reef depth ranges around the MHI, as mesophotic reefs can extend to > 100 m. The MHIA data are saved as snapshots every three hours, while the STRs recorded temperature data every 5 min. In order to compare, the STR values were averaged over the intervals + /- 10 min around each 3-hourly MHIA record (5 measurements). Some STR data recovered in 2013 were only recorded every 30 min, so these were averaged in intervals ± 1 h around each 3-hourly MHIA record (5 measurements) to match the MHIA temporal resolution. Here, we assume that STR temperature measurements reflect true water temperatures against which to evaluate MHIA skill.

MHIA output served from PacIOOS was interpolated from the native ROMS grid onto fixed depth levels and provides modeled temperature across 36 depth strata from the surface to 5500 m at 3-h intervals from 2007 to 2017 at approximately 4-km spatial resolution in the study domain. While the original model has a 450-s time step, only 3-hourly snapshots (*i.e.,* not averages) of the output are saved for storage purposes. Our validation study focuses on subsurface temperature outputs within the depth range of STR loggers (MHIA strata at 5, 10, 20, and 30 m). MHIA temperature data were extracted from the PacIOOS ERDDAP server (https://pae-paha.pacioos.hawaii.edu/erddap/; accessed on 17 Mar 2023) and matched with STR times and locations (averaged across 3-h intervals) using the ‘rerddap’ package in R (version 1.0.0^13^). External data assimilated into the MHIA temperature simulations did not include in situ observations from STRs.

### Quantitative evaluations of MHIA model performance

For an overall comparison between temperature measurements, we used a Deming regression (‘SimplyAgree’ package in R^14^ to estimate the linear relationship between MHIA and STR temperatures as follows:

$$MHIA= \alpha (STR)+ \beta $$

A Deming regression is similar to a simple linear regression but is useful in cases where both variables contain measurement error^15,16^, such is the case with both STR and MHIA temperatures. This regression minimizes the sum of distances in both variables to find the line of best fit while accounting for the error ratio ($\uplambda$) between the two datasets, which was estimated from the coefficients of variation (CV) as follows:

$$\uplambda = \frac{{{(CV}_{MHIA}*\overline{MHIA })}^{2}}{{{(CV}_{STR}*\overline{STR })}^{2}}$$

where $\overline{MHIA }$ and $\overline{STR }$ represent the mean MHIA and STR values. To further assess the model’s skill in representing observed subsurface temperatures, we employed a series of quantitative metrics for pairwise comparisons^17–19^:

Correlation coefficient (r):

1 $$r= \frac{{\sum }_{i=1}^{n}({STR}_{i}-\overline{STR })({MHIA}_{i}-\overline{MHIA })}{\sqrt{\sum_{i=1}^{n}{({STR}_{i}-\overline{STR })}^{2}\sum_{i=1}^{n}{({MHIA}_{i}-\overline{MHIA })}^{2}}}$$

Bias (mean error):

2 $$Bias= \frac{{\sum }_{i=1}^{n}{(MHIA}_{i}-{STR}_{i})}{n}$$

Mean absolute error:

3 $$Absolute error= \frac{{\sum }_{i=1}^{n}{|MHIA}_{i}-{STR}_{i}|}{n}$$

Root mean squared error (RMSE):

4 $$RMSE= \sqrt{\frac{{\sum }_{i=1}^{n}{{(MHIA}_{i}-{STR}_{i})}^{2}}{n}}$$

In the equations above, $n$ represents the total number of STR-MHIA pairs, ${STR}_{i}$ and ${MHIA}_{i}$ represent the $i$th STR observation or MHIA value, and $\overline{STR }$ and $\overline{MHIA }$ represent the mean STR observation or MHIA value. The correlation coefficient (r) measures the strength of correlation, or the degree to which changes in observed values predict changes to the modeled values. The mean error, absolute error, and root mean squared error (RMSE) all measure the magnitude of the differences between estimated and observed values. Mean error is a measure of the model bias, indicating the directionality of discrepancies between estimations and observations. The absolute error and RMSE measure the total magnitude of the error, rather than the direction of these discrepancies.

In addition to calculating these broad scale metrics of overall model performance, we used Taylor diagrams to succinctly visualize the extent of pattern correspondence between modeled and observed temperatures on 2D plots^20^. Taylor diagrams relay several statistics, including the correlation, RMSE, and the ratio of normalized standard deviations of the modeled and observed data, allowing for a quick summary of these metrics across various spatial and temporal scales.

While Taylor diagrams provide an evaluation of model comparison across individual spatial or temporal scales, they do not address the additive effects of these potential biases on the model’s ability to accurately estimate observed data. Thus, we used a generalized additive modeling (GAM) framework to assess the potential spatial, temporal, and environmental biases of MHIA skill. GAMs combine properties of generalized linear models with additive models and allow for the incorporation of nonlinear relationships between predictor and response variables^21^. Using the ‘mgcv’ package in R^22^, we modeled MHIA bias (i.e., the difference between predicted and observed temperatures) as a function of the following predictors: depth (m), distance to shore (m), year, day of year, and location coordinates. The general formula for this nonparametric regression is:

5 $$g\left(\mu \left(x\right)\right)= \alpha + {\sum }_{i=1}^{p}{f}_{i}{(x}_{i})$$

where $\alpha$ is the intercept term, ${f}_{i}$ is the $i$th smoothing function, and ${x}_{i}$ is the $i$th predictor variable. The response variable, $\mu \left(x\right)$, is related to the predictors via a link function, $g()$. Using MHIA bias as the response variable, we fit a GAM with a Gaussian family (identity link) to evaluate how much the predictors listed above account for variance in the model error. Depth and distance to shore were fit using a cubic spline smooth function, day of year was fit using a cyclic cubic spline smooth function, year was fit as a categorical variable, and latitude and longitude were included as a tensor product, allowing these components to be weighted evenly. The few months of data prior to 2011 were excluded from the Taylor Diagrams and GAM analysis as this was the initial set up of the MHIA reanalysis and is considered spin up time.

### Assessment of MHIA performance for coral reef management

Ocean temperature is closely linked to coral health; therefore, it is important that ocean models such as MHIA are able to capture the temperature variability reflected in in situ data in order to identify potential heat stress events on reef ecosystems. We therefore evaluated how accurately MHIA represents this variability across several temperature metrics used to identify heat stress^5^. In particular, we assessed how well MHIA reflects monthly temperature anomalies, and thus its ability to capture extreme heat or cold events, observed from STR data. To do so, we calculated the monthly climatological temperatures from observed and modeled datasets between 2010 and 2017. We then subtracted these monthly climatological temperatures from mean monthly temperatures within the respective datasets and compared the anomalies reflected in observed and modeled temperatures. In addition to monthly anomalies, we further evaluated how well MHIA reflects daily anomalies from the seasonal trend. To do so, we used Generalized Additive Models (GAMs) with daily temperatures fit as a function of day-of-year (cyclic cubic spline) to identify the seasonal trend from the MHIA and STR datasets (r^2^ = 0.73 and 0.70, respectively). We then used the residuals from each of these models as daily detrended temperatures, or temperatures with the seasonal trend removed.

High seawater temperatures alone do not account for all bleaching events. At marginal heat stress temperatures, studies have shown that high temperature variance on short timescales (i.e., biweekly) can also be an indicator and predictor of coral bleaching^23^. To describe this variance, the coefficient of variation (CV) is used to estimate temperature variance independent of the mean. Accordingly, we calculated 14-day temperature means and their associated CV throughout the MHIA and STR datasets and compared them using linear regressions to evaluate how well MHIA captures these heat stress metrics.

Another method with which to measure variability is to apply a power spectral density (PSD) analysis to the time series temperature data that identifies the fundamental periodicities present within a time series (e.g. diurnal cycles, tides). We used PSD analysis to compare the periodicities present within the observed and modeled temperature data. For each dataset, we transformed frequency using the non-parametric Daniell-Kernal method to calculate smooth spectral density via centered moving averages^5,24^. We then generated periodograms for each dataset using a fast Fourier transform^25^, and converted the frequency axis of the periodograms to cycles per unit time by dividing the PSD frequency by the length of the sampling interval (3 h). Finally, we multiplied the spectral density by two so that the area under the periodograms curve matched the time series variance.

## Results

### MHIA skill assessment

**Figure 2:**
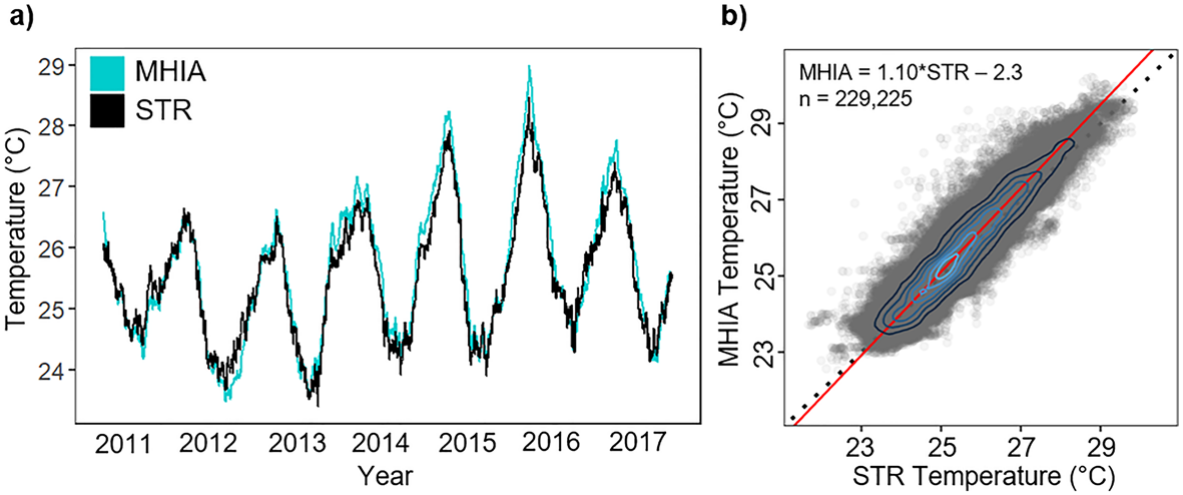
Raw subsurface temperature comparisons. (**a**) Daily subsurface temperature time series from MHIA (teal) and STR (black) datasets averaged across all sites. (**b**) Deming regression of 3-hourly MHIA (modeled) and STR (observed) subsurface temperatures across the MHI for the full time series (2010–2017). The red line indicates the fitted linear trend and the black dashed line represents the 1:1 line. Blue contours indicate density of points (light = high density).

We matched 229,225 subsurface temperature measurements with the MHIA reanalysis across the coastal MHI region. Raw temperature time series showed strong similarities between measured and modeled temperatures (Fig. 2a). The Deming regression revealed a significant positive relationship between overall STR and MHIA temperatures (p < 0.001; Fig. 2b), with MHIA slightly overestimating high temperatures and slightly underestimating low temperatures.

**Figure 3:**
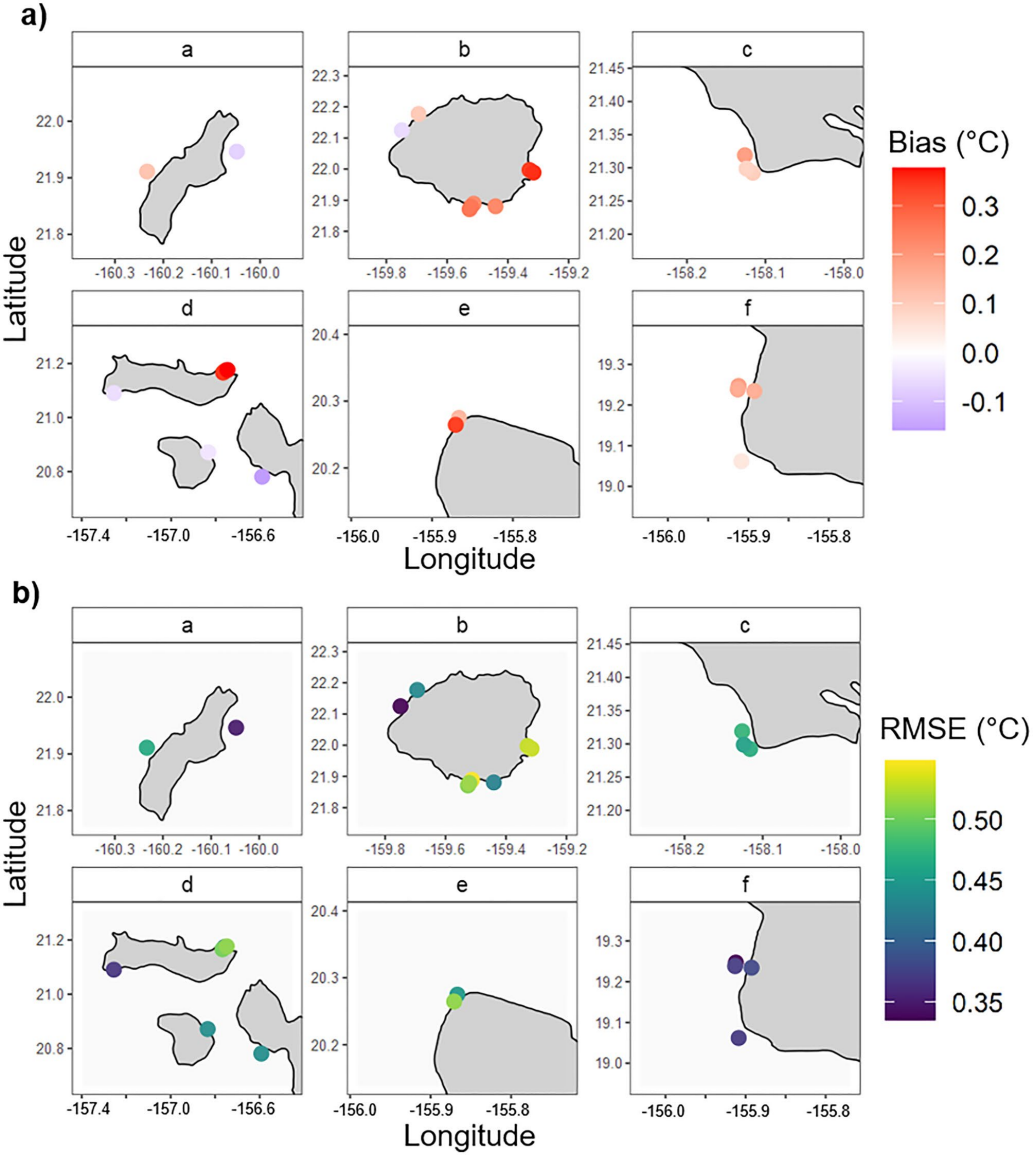
Maps of (**a**) mean MHIA bias and (**b**) root mean squared error (RMSE) for each STR location across the MHI. Units are °C. Lettered panels correspond to boxes in Fig. 1.

The pairwise quantitative skill metrics further showed a very small difference between observed and modeled temperatures (Table 1), all suggesting that the MHIA model performed very well overall. At each STR site, the bias ranged from -0.16 to 0.38 °C (Fig. 3a) and the RMSE ranged from 0.33 to 0.55 °C (Fig. 3b).

**Table 1:** Summary of quantitative statistics for evaluating overall MHIA model skill in relation to subsurface temperature measurements.

Quantitative skill metrics	
Correlation (r)	0.94
Bias	0.17 °C
Mean absolute error	0.35 °C
Root mean squared error (RMSE)	0.45 °C

**Figure 4:**
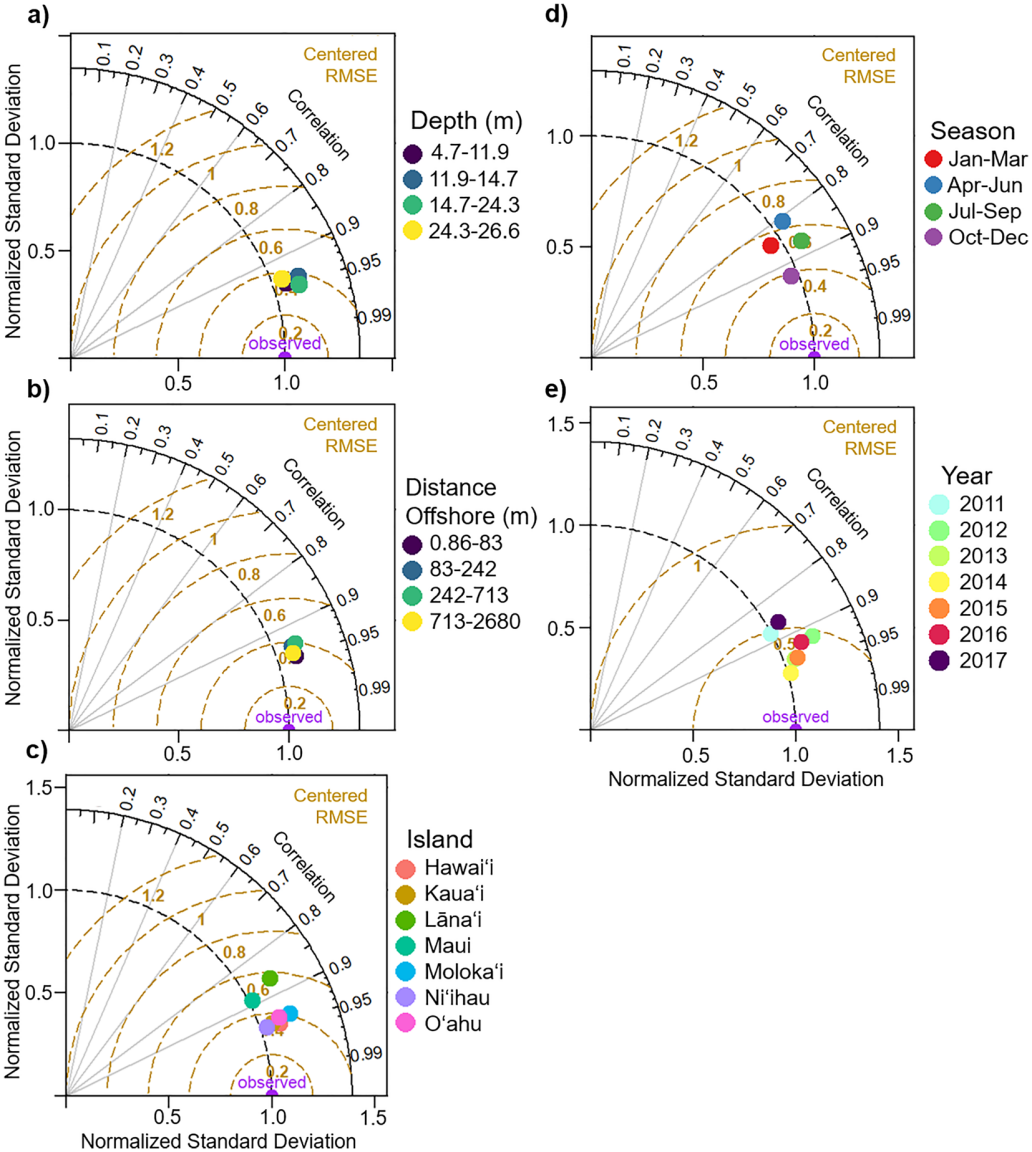
Normalized Taylor diagrams for (**a**) depth, (**b**) distance offshore, (**c**) island, (**d**) season, and (**e**) year. The “observed” point on the x-axis represents the normalized observation, and modeled data lying nearest to this point agree well with observations. This point indicates high correlation (outer arc), low RMSE (gold arcs), and equal variability (dashed arc) between modeled and observed data.

Taylor diagrams provided several metrics to evaluate the skill of MHIA (Fig. 4). These diagrams show the correlation (outer black arc) and RMSE (inner gold arcs) between observed and modeled temperatures, and indicate how well the modeled temperature variability matches that of the observed temperature variability (standard deviation; dashed arc). The “observed” points on the x-axis represent the normalized observation, and modeled data lying nearest to this point agree well with observations. The diagrams revealed that MHIA performed very well across spatial scales (depth, distance from shore, island), with high correlation (r > 0.85), low RMSE (RMSE < 0.6 °C), and similar variability in modeled and observed data (Fig. 4a–c). Lānaʻi and Maui had slightly lower correlation and higher RMSE than the other islands, likely due to having the fewest STR sites, while Molokaiʻi and Niʻihau had the most similar variability between modeled and observed data. MHIA performance was similarly high across temporal scales (season, year; Fig. 4d, e). Across seasons, Oct-Dec performed slightly better than all other seasons (r = 0.94, RMSE = 0.4). Across years, 2011 and 2017 had slightly lower correlation and RMSE than other years, while variability in modeled compared to observed data differed most in 2012. MHIA performed well during Hawaiʻi’s recent coral bleaching years (2014 and 2015).

**Figure 5:**
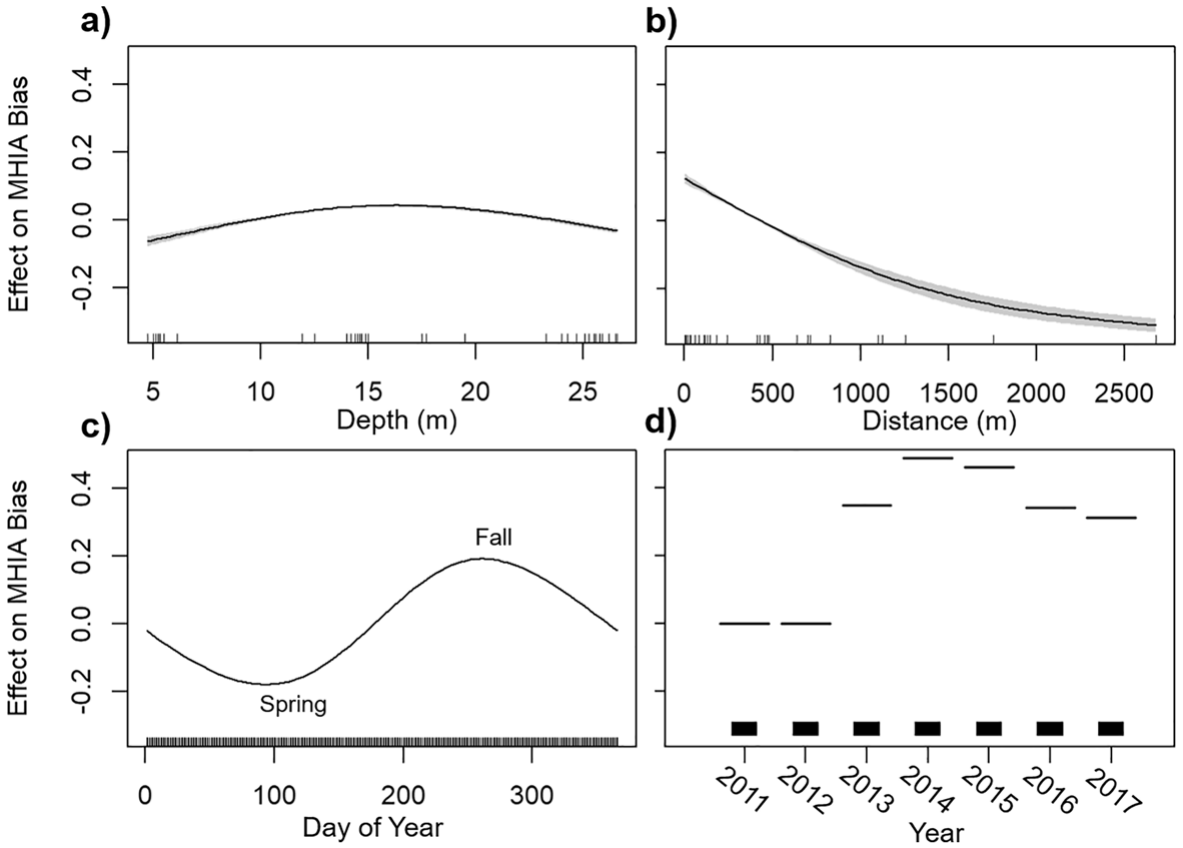
Results from the generalized additive model (GAM) showing partial effects of spatial, temporal, and environmental variables on MHIA bias: (**a**) depth, (**b**) distance offshore, (**c**) day of year, and (**d**) year. Solid lines represent the smoothed model fits and grey shaded regions show the 95% confidence intervals. In (**d**), 2011 was used as a baseline to which all other years are compared.

The GAM results additionally uncovered minor spatial and temporal biases in MHIA error, though the partial effect of any given predictor did not influence the error more than ± 0.45 °C. The GAM explained 25% of the total deviance in MHIA bias and all predictors were found to be significant (p < 0.001). When accounting for the additive effects of all predictors, MHIA appeared to perform well across depths (Fig. 5a) but slightly underestimated temperatures at greater distances from shore (Fig. 5b). Seasonally, MHIA slightly underestimated temperatures during the late winter and early spring and slightly overestimated during the early fall (Fig. 5c). Across years, MHIA had the greatest positive biases in 2014 and 2015, respectively (Fig. 5d).

### Evaluation of heat stress metrics

**Figure 6:**
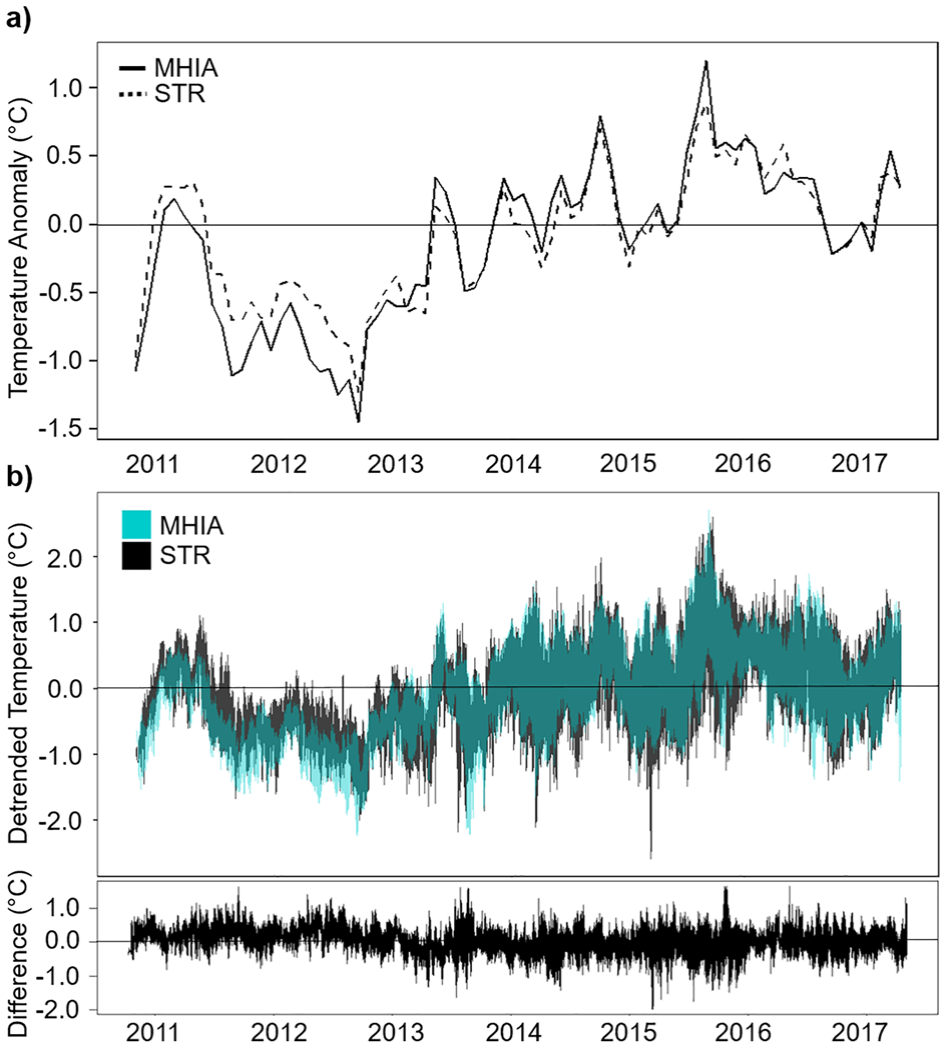
Subsurface temperature anomalies. (**a**) Monthly temperature anomalies calculated for the MHIA (solid) and STR (dashed) time series. Anomalies were calculated using 2010–2017 climatologies for each dataset. **b)** Daily temperature anomalies from the seasonal trend (i.e., detrended temperatures) calculated for the MHIA (teal) and STR (black) time series. The bottom panel shows the daily differences (observed–modelled) between detrended temperatures.

A comparison of monthly temperature anomalies between the observed and modeled temperatures showed very similar temporal trends (Fig. 6a). MHIA slightly underestimated positive temperature anomalies and estimated lower than observed negative anomalies prior to 2013. During the fall of 2015, MHIA slightly overestimated a strongly positive temperature anomaly. Using the estimates from^11^, we calculate average MHIA uncertainty for the upper 30 m of all the coastal temperatures to be 0.28 °C. Therefore, the differences between modeled and observed monthly anomalies throughout the time series are within the bounds of estimated error. Comparisons of daily detrended temperatures between the MHIA and STR temperatures likewise showed similar temporal trends (Fig. 6b). Several anomalously cold days that were observed in 2013, 2014, and 2015 were not reflected in the MHIA temperatures, and several observed days in 2013, 2015, and 2017 were estimated to be more anomalously cold by the model than was observed by STR data. However, the vast majority of daily anomalies were well represented by the model and the differences between MHIA and STR temperatures were largely less than 0.5 °C.

**Figure 7:**
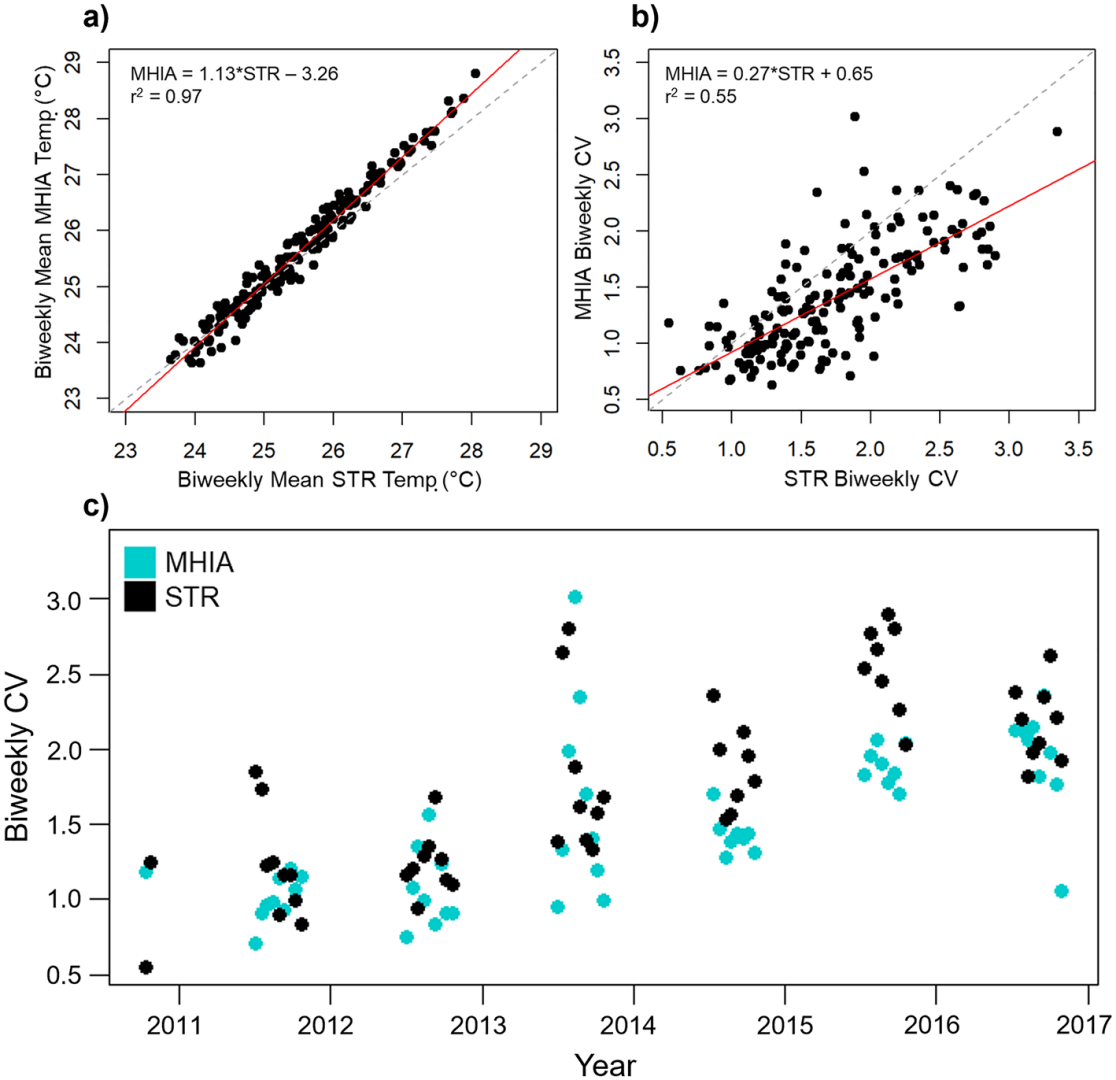
Linear regressions for MHIA (modeled) and STR (observed) biweekly temperature (**a**) means and (**b**) coefficients of variance (CV) across all STR depths for the full MHI time series (2010–2017). The dashed line represents the 1:1 line and the red line indicates the fitted linear trend. (**c**) Time series of biweekly CV during peak bleaching times (Jul–Oct) calculated from MHIA estimates and STR measurements.

Our evaluation of 14-day temperature metrics indicated that MHIA estimated mean subsurface temperatures across this time window nearly identical with those observed from in situ measurements at all depths (r^2^ = 0.97; Fig. 7a). However, MHIA largely underestimated biweekly CV values (r^2^ = 0.55; Fig. 7b), indicating that it did not estimate variance at biweekly timescales as well. The trend in biweekly CV across the nearshore MHI region increased annually throughout the time series in both STR and MHIA estimates, though MHIA estimates of this metric were usually slightly lower than STR estimates (Fig. 7c).

**Figure 8:**
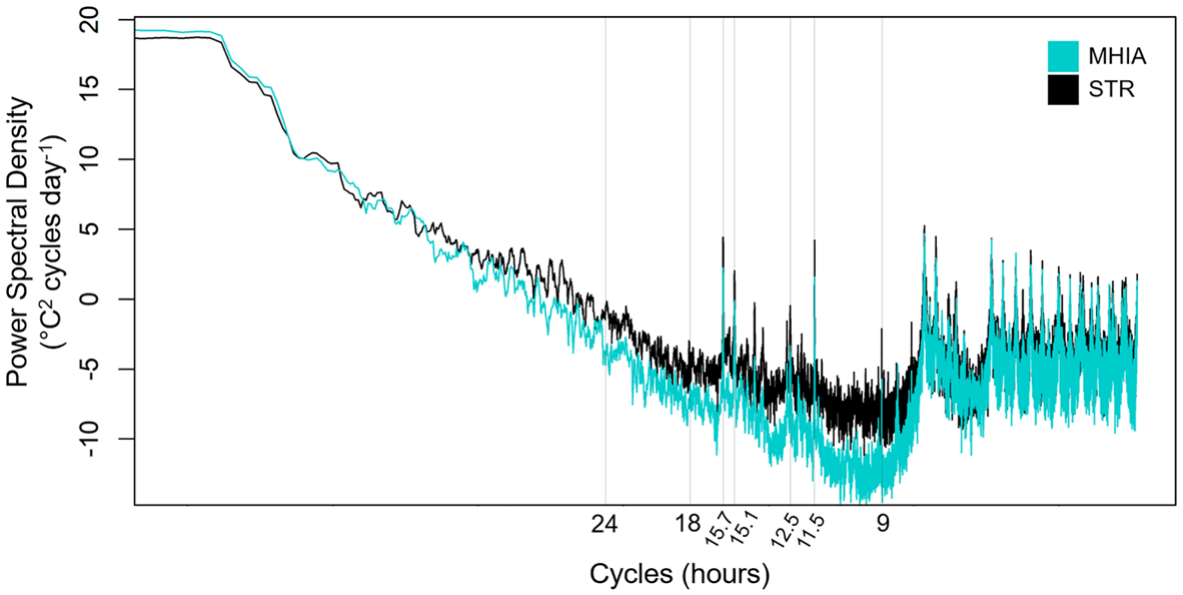
Power Spectral Density (PSD) results from the STR (black) and MHIA (teal) temperature time series presented in a periodogram. Peaks in PSD indicate cycles of temperature variability at the time cycles labeled on the x-axis.

The PSD analysis showed that MHIA estimated the same cycles of variability throughout the temperature time series as the observed data (Fig. 8). Peaks in the periodogram, representing high temperature variation, were observed in both datasets at 9, 11.5, 12.5, 15.1, 15.7, 18, and 24 h. While the original MHIA model resolves periods as low as 15 min, with 3-hourly output, we can only present periods as low as 6-hourly. As shown in Fig. 8, the MHIA captures the same periods of elevated energy as identified by the STR; however, the MHIA has lower energy in the 8–18 h bands (except at the semidiurnal tides that are similar in energy). As there are a number of dynamics that can influence temperatures at these frequencies, one major reason for this energy difference is wind variability. The MHIA is run with 3-hourly winds, which would suppress coastal wind variability that would alter winds at these higher frequencies.

## Discussion

Accurately monitoring temperatures at depth is critical for understanding heat stress on coral reefs and predicting bleaching events. The temperatures and variability experienced by corals can be very different from sea surface values measured from orbit, and deeper corals may be adapted to lower temperatures or reduced variability compared to those near the surface^26^. In addition to the MHIA ocean circulation model, there are also remote-sensing observations, particularly from satellite SST. NOAA Coral Reef Watch (CRW) provides 5 km blended satellite SST products for coral reef research that have been crucial to the development of coral heat stress metrics. We compared CRW SST measurements to the in situ temperature data from STRs and found that the comparisons were similarly robust to the model estimates (r = 0.96; bias = 0.08 °C; RMSE = 0.32 °C). However, SST does not provide additional subsurface information from the data loggers; whereas, the model provides the temporal and spatial structure of the ocean that is responsible for the temperatures observed. Satellite SST products alone may not reflect the most relevant temperature metrics for corals. Thus, it is essential to supplement SST with reliable and high-resolution subsurface temperature data in order to manage these increasingly vulnerable ecosystems.

### MHIA accurately estimates subsurface temperatures

The MHI region is influenced by dynamic oceanography. The westward North Equatorial Current flows around the island chain after encountering the island of Hawaiʻi, while mesoscale eddies and currents form in the lee of the islands as consistent northeast trade winds interact with island topography^27,28^. MHIA was designed to capture these offshore dynamics with a 4 km horizontal resolution. Each grid cell may represent a volume of 160 million liters as compared to a point observation from instruments such as STR that sample around 1 L. Despite the model representing a much larger volume than the STR, this study finds that the model performs very well at shallow nearshore sites (well within the analysis error of the model) and estimates of the subsurface temperatures to ~ 30 m depth compare well around the MHI. This is partially because Hawaiʻi and other Pacific Islands are uniquely exposed to open ocean conditions and more directly influenced by offshore dynamics.

MHIA temperatures were highly correlated with STR data and had minimal overall bias (< 0.2 °C) across all sites. By comparison, a skill assessment of a ROMS configuration in the Barents Sea found subsurface temperature biases on the order of 0.5°C^12^, and an assessment of the Finite Volume Community Ocean Model (FVCOM) in the Northwest Atlantic Shelf found an overall temperature bias of 0.04°C^18^. However, these studies evaluated temperatures down to several hundred meters. Accordingly, a future expansion of the analyses presented here should aim to validate deeper depths across Hawaiʻi as well as other U.S. Pacific Islands where PacIOOS ROMS operates (e.g.*,* Guam, American Samoa).

One limitation of assessing MHIA temperature estimations against subsurface measurements is that we rely on the assumption that the STR measurements represent true in situ temperatures. Some temperature loggers have instrumental drift due to biofouling, as they are left untouched for three years at a time. Nevertheless, the temperature accuracy of the STRs is ± 0.002 °C, which is substantially better than that of the MHIA model or SST, and the instruments were calibrated before and after deployment to assess potential drift (< 0.002 °C year^−1^). The STRs provide the most robust time series of subsurface temperature measurements within MHI coral reef habitats and allowed us to conduct a thorough skill assessment of modeled temperature outputs.

### Modeled temperatures capture most, but not all, variability in observed data

MHIA temperature estimates appeared to be robust across spatial (*i.e.,* depth, distance from shore, island) and temporal (i.e., season, year) scales. The Taylor diagrams suggest that modeled temperatures were slightly more representative of observations during the fall (Oct-Dec; Fig. 4d), while the GAM results suggest that MHIA were closest to observations during the late spring (Fig. 5d). Late summer and early fall temperatures (Aug-Oct) were slightly overestimated by MHIA according to the GAM. Temperature accuracy during these months is most critical as coral stress and bleaching potential is greatest during the summer-fall, and while seasonal differences in MHIA estimation accuracy were minimal, they are important to consider.

The GAM results indicate that 2014 and 2015 had the highest positive MHIA bias with respect to the 2011 baseline. 2015 also had the highest positive subsurface temperature anomaly in our time series (Fig. 6a), which indicates that MHIA slightly overestimated this strong temperature anomaly. Warm SST anomalies have occurred around Hawaiʻi since 2013, but in 2015 Hawaiʻi experienced record-breaking summer temperatures and the highest positive SST anomalies observed in decades^29^. Thus, it appears that MHIA may slightly overestimate the extent of extreme warm events. However, one would expect errors in the MHIA temperature estimates on the order of > 0.28 °C given that each STR point is matched with a MHIA output roughly 160 million times greater in volume. Therefore, the differences between modeled and observed temperature anomalies were always within the uncertainty bounds of the estimations and the two time series cannot be viewed as significantly different. At the daily scale, MHIA did not reflect the coldest temperature anomalies in the time series (Fig. 6b). This suggests that the model may be limited in capturing short-lived cold events that can likewise have negative impacts to reef health^30,31^. Nevertheless, the majority of daily anomalies were well represented by the model and differences between the detrended temperature datasets were generally within the uncertainty bounds of the estimations.

Overall, 3-hourly MHIA temperatures were well matched with in situ temperatures throughout the time series (Fig. 2b). At timescales relevant to coral bleaching (i.e., biweekly), MHIA subsurface temperatures likewise represented in situ temperatures very well (Fig. 7a). However, modeled temperature variability was underestimated at this time scale (Fig. 7b,c), suggesting that biweekly CV may not be as reliable as other heat stress metrics represented by MHIA. Accordingly, biweekly CV stood out as particularly high in the STR dataset (beyond the generally increasing trend) during the summer and early fall of 2015 when Hawaiʻi experienced record high seawater temperatures, but did not stand out beyond the increasing trend in the MHIA estimates. Thus, MHIA underestimates biweekly CV as produced by the observations, so by this metric the model will be more conservative than the observations in estimating potential bleaching conditions. Still, this variance was significantly positively correlated between modeled and observed data and is likely useful.

We also found that MHIA reflected cycles of temperature variability throughout the time series accurately, though for a band of frequencies at a lower energy as observed in STR data (Fig. 8). Subsurface temperatures are affected by solar heating (and nighttime cooling), mixing, eddies, and upwelling at varying spatiotemporal scales. Hawaiʻi’s mixed-semidiurnal tides have a strong influence on nearshore subsurface temperature, which generally have changes in ebb and flow every ~ 6 h. Internal waves, which are generated around Hawaiʻi from interactions between tidal currents, stratification, and underwater topography^32^, also have a strong effect on temperatures around the MHI. The dominant internal tide around the MHI is M_2_ (12.42 h), which is revealed by both STR and MHIA. The diurnal tides do not produce significant internal tides around the islands as shown by very little energy at the 24-h period.

### MHIA as a tool for coral reef management

Obtaining high resolution temperature data at the depths and locations of vulnerable corals is essential for the management and conservation of reef ecosystems. But, monitoring the thermal environment of every reef at every time point would require significant time and resources to maintain and is largely infeasible. We have evaluated the ability of MHIA to recreate these subsurface temperatures across Hawaiʻi nearshore habitats and whether it can be used to complement or replace in situ data as needed. For assessing mean temperature conditions at various timescales, the model appears to be very accurate and provides robust estimates of the general thermal regime across the nearshore MHI. MHIA has very minimal errors with respect to temperature loggers (generally much less than 1 °C), accurately reflects monthly temperature anomalies, and largely captures the same cycles of variability observed from subsurface measurements. This suggests that MHIA may be useful for tracking potential coral bleaching conditions using several of the commonly employed heat stress metrics. It is important to note that very shallow reefs can experience significant coral bleaching due to high temperature variability in surface waters, and our study is limited to evaluating the utility of MHIA for reef systems greater than ~ 5 m.

There are some heat stress metrics that are not reproduced as robustly by the model, including biweekly CV as an indicator of variability. However, there is a significant positive correlation (r^2^ = 0.4) between estimated and observed CV, suggesting that there may still be some utility in calculating this metric from MHIA. The impact of temperature variance on coral bleaching prevalence can further be evaluated from PSD analyses. While MHIA captures the same cycles of temperature variability as those detected in measurements, the model does not represent the same amount of energy outside of the dominant peaks. This suggests that fine-scale (*i.e.,* sub-daily, daily) temperature variance calculated from the model’s 3-hourly outputs may not always reflect true conditions when trying to correlate these cycles with bleaching events. Consequently, heat stress metrics that focus on temperature variability across shorter time scales may not be as consistently represented by modeled temperatures. High-frequency cooling events such as internal bores also play an important role in ameliorating thermal stress to corals, as has been found in MHI shallow coral reefs and elsewhere^33,34^. While these events are captured by the STRs, they are not reflected in the 3-hourly estimates produced by MHIA.

**Figure 9:**
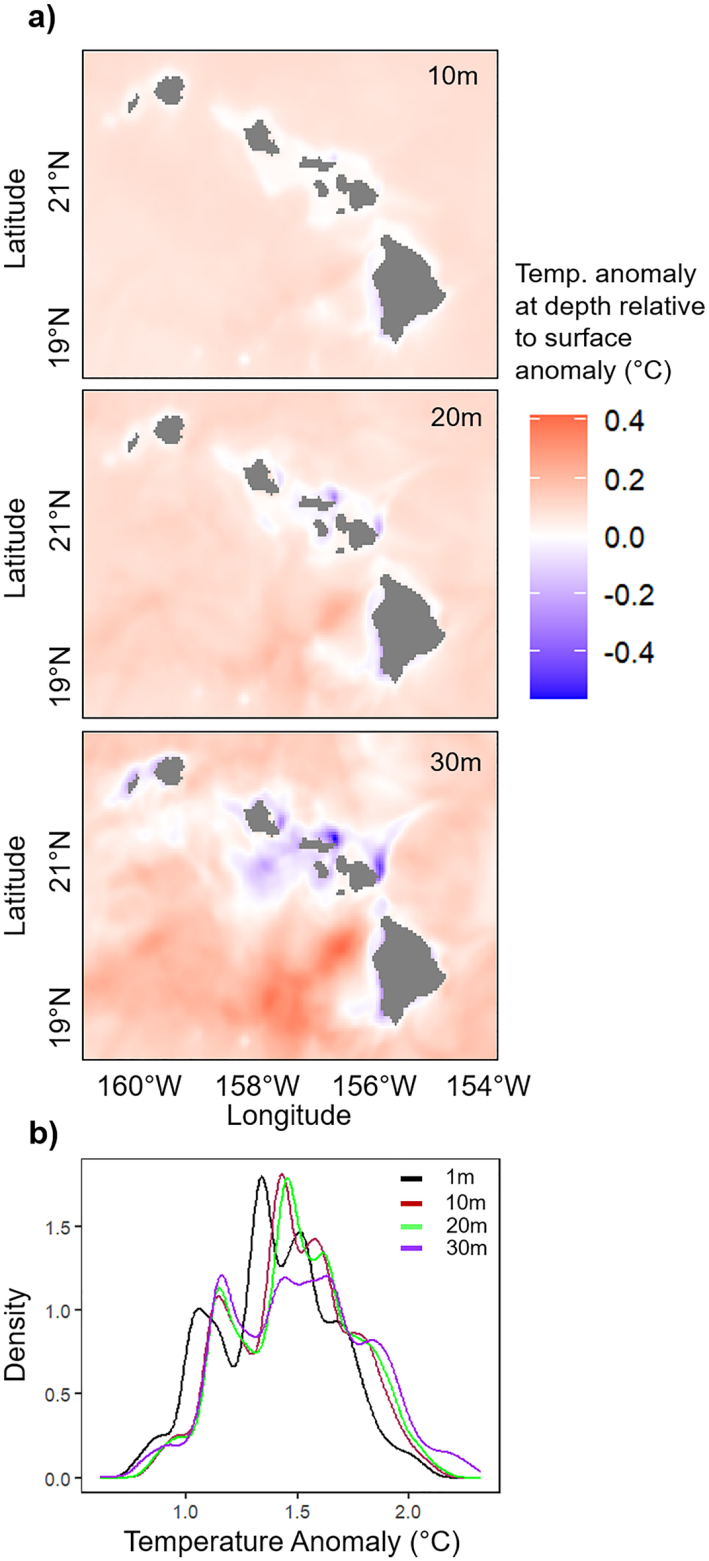
Modeled heat signals during the 2015 bleaching event. (**a**) Maps showing the difference between MHIA temperature anomalies at 10 m, 20 m, and 30 m relative to surface temperature anomalies for September 2015. Anomalies for September 2015 were calculated from within the timeframe evaluated in this study (2010–2017). (**b)** Density plot showing the differences in temperature anomalies across depths for September 2015.

Hawaiʻi experienced major coral bleaching in 2014 and 2015^35,36^ as one of many regions influenced by a global bleaching event from 2014 to 2017^37^. Importantly, both STR and MHIA temperature data identified the same months that had anomalously high temperatures in 2014 and 2015 (Fig. 6a). These matched the bleaching and SST anomalies around Hawaiʻi that peaked during the summer and fall months of those years^2,29^. Thus, for stress metrics like heat accumulation over longer time scales (i.e., weeks, months), MHIA may be useful for monitoring potential bleaching conditions in nearshore MHI waters. These data could directly be used for management, such as by pairing subsurface MHIA temperatures and thermal stress metrics with long-term monitoring data to identify areas where corals are particularly vulnerable or resilient to warming. The data may also help identify thermal refugia where restoration efforts can be prioritized. While satellite-derived SST provides a robust estimate of the subsurface temperatures recorded by STR around Hawaiʻi, MHIA provides environmental data (temperature, salinity, velocity) at all reef depths. MHIA has the ability to identify potential differences between surface and subsurface temperatures, which is particularly useful for coral reef management and in regions where reef habitats are not well mixed with surface temperatures. By providing a more detailed understanding of the coral reef relevant thermal environment, MHIA can aid in predicting and mitigating the impacts of climate change, such as coral bleaching events. During the 2015 bleaching event across the MHI, heat signals that differed across depths can be visualized from the model but are not discernable from surface satellite measurements (Fig. 9).

Overall, MHIA demonstrates high accuracy across the nearshore MHI region and captures a significant amount of observed subsurface temperature variability. This is partially due to the exposed open ocean conditions and more direct influence of offshore dynamics experienced by Pacific Island regions. As such, MHIA temperature estimates prove to be a useful tool for coral reef management in the absence of, or to supplement, subsurface measurements across Hawaiʻi and likely for other Pacific Island regions.

### Supplementary Information

Supplementary Table S1.

## Data Availability

The STR data used in this study is publicly available via https://www.ncei.noaa.gov/erddap/tabledap/CRCP_Subsurface_Temp_Hawaii.html and the ROMS temperature reanalysis data is publicly available via https://pae-paha.pacioos.hawaii.edu/erddap/griddap/roms_hiig_reanalysis.html. R code is available via https://github.com/jnperelm/MHI_ROMSval.
