# Supplementary material for: Subsurface temperature estimates from a Regional Ocean Modelling System (ROMS) reanalysis provide accurate coral heat stress indices across the Main Hawaiian Islands
Source: Sci Rep. 2024 Mar 19;14:6620. doi: 10.1038/s41598-024-56865-x (PMC10951325; doi:10.1038/s41598-024-56865-x)
Supplement: Supplementary file 1 — Supplementary Table S1. [file 41598_2024_56865_MOESM1_ESM.docx]

**Supplementary Materials:**

**Subsurface temperature estimates from a Regional Ocean Modelling System (ROMS) reanalysis provide accurate coral heat stress indices across the Main Hawaiian Islands**

**Table S1.** Locations and depths of each STR logger around the Main Hawaiian Islands.

| **STR Site ID** | **Longitude** | **Latitude** | **Island** | **Depth (m)** |
| --- | --- | --- | --- | --- |
| OCC-HAW-001 | -155.86099 | 20.27025 | Hawaiʻi | 25 |
| OCC-HAW-002 | -155.86041 | 20.26826 | Hawaiʻi | 15 |
| OCC-HAW-009 | -155.90043 | 19.24421 | Hawaiʻi | 25 |
| OCC-HAW-010 | -155.90028 | 19.24434 | Hawaiʻi | 15 |
| OCC-HAW-011 | -155.89974 | 19.24447 | Hawaiʻi | 5 |
| OCC-HAW-017 | -155.90162 | 19.07381 | Hawaiʻi | 15 |
| OCC-KAU-001 | -159.32790 | 21.99812 | Kauaʻi | 25 |
| OCC-KAU-002 | -159.32854 | 21.99845 | Kauaʻi | 15 |
| OCC-KAU-003 | -159.52532 | 21.87574 | Kauaʻi | 25 |
| OCC-KAU-004 | -159.52550 | 21.87666 | Kauaʻi | 15 |
| OCC-KAU-005 | -159.52549 | 21.88224 | Kauaʻi | 5 |
| OCC-KAU-006 | -159.68715 | 22.17148 | Kauaʻi | 25 |
| OCC-KAU-010 | -159.44746 | 21.86885 | Kauaʻi | 5 |
| OCC-KAU-011 | -159.76134 | 22.13801 | Kauaʻi | 15 |
| OCC-LAN-007 | -156.83466 | 20.87053 | Lānaʻi | 15 |
| OCC-MAI-011 | -156.58445 | 20.79081 | Maui | 15 |
| OCC-MOL-002 | -156.76055 | 21.17865 | Molokaʻi | 25 |
| OCC-MOL-003 | -156.76049 | 21.17693 | Molokaʻi | 15 |
| OCC-MOL-004 | -156.76071 | 21.17579 | Molokaʻi | 5 |
| OCC-MOL-011 | -157.26696 | 21.08093 | Molokaʻi | 15 |
| OCC-NII-002 | -160.23764 | 21.90168 | Niʻihau | 25 |
| OCC-NII-005 | -160.06176 | 21.95129 | Niʻihau | 15 |
| OCC-OAH-013 | -158.12658 | 21.30575 | Oʻahu | 25 |
| OCC-OAH-014 | -158.12623 | 21.30563 | Oʻahu | 15 |
| OCC-OAH-015 | -158.11841 | 21.30949 | Oʻahu | 5 |
